# Somatic mutation detection efficiency in *EGFR*: a comparison between high resolution melting analysis and Sanger sequencing

**DOI:** 10.1186/s12885-020-07411-1

**Published:** 2020-09-22

**Authors:** Reenu Anne Joy, Sukrishna Kamalasanan Thelakkattusserry, Narendranath Vikkath, Renjitha Bhaskaran, Sajitha Krishnan, Damodaran Vasudevan, Prasanth S. Ariyannur

**Affiliations:** 1 Molecular Oncology Diagnostics Laboratory, Amrita Institute of Medical Sciences, Amrita Vishwa Vidyapeetham, Kochi, 682041 India; 2 Department of Biostatistics, Amrita School of Medicine, Amrita Vishwa Vidyapeetham, Kochi, 682041 India; 3 Department of Biochemistry, Amrita School of Medicine, Amrita Institute of Medical Sciences, Amrita Vishwa Vidyapeetham, Ponekkara P.O., Kochi, Kerala 682041 India

**Keywords:** Sanger sequencing, High resolution melting analysis, Analytical validation, Limit of detection, Somatic mutation, EGFR

## Abstract

**Background:**

High resolution melting curve analysis is a cost-effective rapid screening method for detection of somatic gene mutation. The performance characteristics of this technique has been explored previously, however, analytical parameters such as limit of detection of mutant allele fraction and total concentration of DNA, have not been addressed. The current study focuses on comparing the mutation detection efficiency of High-Resolution Melt Analysis (HRM) with Sanger Sequencing in somatic mutations of the *EGFR* gene in non-small cell lung cancer.

**Methods:**

The minor allele fraction of somatic mutations was titrated against total DNA concentration using Sanger sequencing and HRM to determine the limit of detection. The mutant and wildtype allele fractions were validated by multiplex allele-specific real-time PCR. Somatic mutation detection efficiency, for exons 19 & 21 of the *EGFR* gene, was compared in 116 formalin fixed paraffin embedded tumor tissues, after screening 275 tumor tissues by Sanger sequencing.

**Results:**

The limit of detection of minor allele fraction of exon 19 mutation was 1% with sequencing, and 0.25% with HRM, whereas for exon 21 mutation, 0.25% MAF was detected using both methods. Multiplex allele-specific real-time PCR revealed that the wildtype DNA did not impede the amplification of mutant allele in mixed DNA assays. All mutation positive samples detected by Sanger sequencing, were also detected by HRM. About 28% cases in exon 19 and 40% in exon 21, detected as mutated in HRM, were not detected by sequencing. Overall, sensitivity and specificity of HRM were found to be 100 and 67% respectively, and the negative predictive value was 100%, while positive predictive value was 80%.

**Conclusion:**

The comparative series study suggests that HRM is a modest initial screening test for somatic mutation detection of *EGFR*, which must further be confirmed by Sanger sequencing. With the modification of annealing temperature of initial PCR, the limit of detection of Sanger sequencing can be improved.

## Background

The molecular changes occurring in cancer cells are large and complex [1]. Recent advances in understanding the molecular mechanisms in cancer cells have shed light into several specific molecular variations deemed to be driving the cancer process and recurrence. One of these specific molecular drivers is the activating mutations in the epidermal growth factor receptor (*EGFR*) gene [2]. These activating mutations reside in the Tyrosine Kinase domain (TKD), located across the coding region of the exons 18, 19, 20, and 21 of the *EGFR* gene. The impact of variations in this gene is of great significance as specific pharmaceutical agents inhibiting the activated, but mutated, EGFRs are now used in routine clinical management of lung cancer with significant progression free survival in several large populations [3–5]. These pharmaceutical compounds, called targeted therapy, specifically bind, and inhibit the spontaneously activated EGFRs expressed by specific cancer cells with altered *EGFR* gene. The somatic activating mutations of *EGFR* gene are most widely seen in non-small cell lung cancers (NSCLC), especially in the TKD, and are routinely screened in all NSCLC [6, 7].

Several molecular diagnostic laboratory methods are currently used for the identification of genomic variation of *EGFR* gene with varying degrees of analytical performance and depending upon the availability of resources, expertise and affordability [8–12]. One such technique is high resolution melting analysis (HRM). HRM has the advantage of detecting minute changes in the melting temperature of a target amplicon due to a variation in its sequence as compared to the wild-type, using a saturating fluorescent dye [10]. HRM, like Sanger sequencing (SEQ), is an unbiased qualitative assay to detect any mutation in the target gene region, unlike allele specific oligonucleotide probes employed in popular real-time PCR based assays. Moreover, HRM can be performed as an additional procedure following an initial PCR at a lower cost compared to direct SEQ.

The clinical utility of HRM analysis in *EGFR* mutation detection has been studied previously [13–16]. The performance characteristics of mutation scanning by HRM over SEQ of *EGFR* gene have been investigated previously by two laboratories, showing mixed performance characteristics [17, 18]. Archived tumor tissues from 37 cases in one study showed that HRM had 90% sensitivity and 100% specificity as compared to direct SEQ. The second study, conducted by Do et al., showed 100% sensitivity, in more than 70 cases, and less than 90% specificity in each of the four different exons. These studies, however, did not address analytical validation parameters such as lowest limit of detection (LOD) in terms of DNA concentration and/or the detection limit of Mutant Allele Fraction (MAF) of somatic variants of *EGFR* gene in varying mixed template DNA concentrations. In order to address the varied performance characteristics, we conducted, in the current study, a series of assays to determine the LOD of total DNA per assay by HRM and SEQ. Subsequently by titrating mutant DNA (mutDNA) against wildtype (wtDNA) controls in a series of controlled mixed DNA assays, we determined the LOD of MAF by both the methods. Validation of the MAFs were done using multiplex allele-specific real-time PCR. We, then, compared the performance characteristics of somatic mutation detection by HRM and SEQ in DNA obtained from 116 formalin fixed paraffin embedded (FFPE) tumor tissue samples.

## Methods

### Sample size

A prospective series study in which consecutive FFPE tumor samples, sent to Molecular Oncology Diagnostics Laboratory (MODL) for SEQ of the *EGFR* gene TKD region and mutation analysis were subjected to HRM. Samples irrespective of the tissue of origin, stage of the disease and demographic characteristics were included in the study. Following the screening of 275 FFPE samples, a total of 116 samples were taken for the comparative study. Pathogenic variations in *EGFR* TKD were identified in 75 out of 275 samples by SEQ. HRM was performed in 67 of the 75 positive samples, and first 49 of the 200 negative samples, in chronological order. Eight positive mutant samples were not included for comparative analysis due to low quantity of DNA.

### Sample preparation

Tumor tissue samples obtained from patients were subjected to grossing and dissection, fixed in neutral buffered formalin solution, embedded in paraffin, sectioned, and stained. The tissues in paraffin blocks (FFPE) were sectioned and subjected to histopathological examination by the pathologists in the Department of Pathology. Following the determination of the histological as well as immunohistochemical characteristics of the tumor, and the subsequent assessment of the adequacy of the cellular content in the sections, region of interest from each tumor tissue was selected to enrich tumor cell content to about 50% for molecular genetic analysis. Approximately 10 sections of the selected regions of each tumor tissue, with a thickness of 10 μm, were obtained in a 1.5 ml tube and transferred to MODL at room temperature for mutation analysis.

The DNA from FFPE sections was extracted using QIAmp® FFPE DNA extraction kit (Qiagen, USA), according to the procedure described by the manufacturer. DNA quantification was performed by checking the absorbance at 260 nm and 280 nm by spectrophotometric analyzer (Thermo Fisher Scientific, USA), and fluorometric method using Qubit3.0® fluorometer (Thermo Fisher Scientific, USA). Downstream processing of the extracted DNA was performed only when the ratio of absorbance at 260/280 was ≥1.8 with the concentration of DNA ≥ 5 ng/μl.

### Initial amplification of DNA

In a 20 μl assay, 1X Emerald GT PCR master mix (Takara/Clontech, USA) was added, along with m13-tagged forward and reverse target primers (5 μM). Approximately 50 ng of template DNA is added, in a typical assay, and made up with distilled water. Primers for exons 18, 19, 20, and 21 of the *EGFR* gene (NCBI Genbank Accession ID: NM_005228.3) were synthesized (Merck-Sigma, Bangalore, India). Design and characterization of the primer sequences for both sequencing and HRM were obtained from a previously published literature [18]. Thermal cycler settings included an initial denaturation of 95 °C for 15 min, followed by 45 cycles of denaturation at 94 °C for 45 s, annealing at 58 °C for 45 s, extension at 72 °C for 45 s and a final extension at 72 °C for 10 min. The amplicons were assessed using 2% Agarose gel (SeaKem® LE Agarose, Lonza, USA). The PCR products were then subjected to post-PCR clean up to remove residual primers and other enzyme proteins using HighPure® PCR product purification kit (Roche Molecular Diagnostics, Switzerland).

### PCR for high resolution melting analysis

In a 20 μl assay, 1X of High-Resolution Master mix (Roche Molecular Diagnostics, Switzerland), 300nΜ each of forward and reverse primers, and 2.5 mM of MgCl_2_ were added. As described in the previous study [18], 5 ng of template DNA was added, and the assay was made up with PCR-grade distilled water. The assay strip tubes were loaded onto the LightCycler480®. The assay was optimized based on previously described method [18] with minor modifications. The standardized thermal cycler settings include - an initial denaturation at 95 °C for 15 min followed by 50 cycles of denaturation at 95 °C for 10 s, annealing at 65 °C for 10 s and extension at 72 °C for 30 s, with initial 10 cycles of touchdown (1 °C /cycle). This is followed by final denaturation at 95 °C for 1 min and cooling at 4 °C for 2 min. The high-resolution melting was performed from 65 °C to 95 °C at a ramp rate of 0.02 °C/s with 25 fluorescence data acquisition points, followed by cooling to 4 °C for 30 s.

### Sanger sequencing

DNA sequencing using Sanger’s dideoxy method was performed using BigDye® Terminator cycle sequencing kit v3.1 compatible with ABI 3500® Genetic analyzer. The original reaction setup, recommended by the manufacture, was optimized with the following modifications. In a total assay volume of 10 μl of separate forward and reverse reactions, BigDye® Terminator, 1X sequencing buffer, 0.8 μM of m13 forward or reverse primer were added to the respective reaction assays. Post-PCR purified DNA amplicon was added, and the volume was made up with distilled water. Assay conditions were setup by the manufacturer recommendations with the following modifications. Initial denaturation at 96 °C for 1 min, 15 cycles of denaturation (at 96 °C for 10 s), annealing (at 50 °C for 5 s) and extension (at 60 °C for 75 s), followed by final extension (2 set of 5 cycles with expanding the extension phase 60 °C to 90 s and 2 min subsequently). The amplified product was stored at 4 °C. Post-sequencing PCR clean-up was done using Sephadex® G-50 medium (molecular weight cut-off of 30,000 Mr) (GE Lifesciences), and Whatman UNIFILTER® filtration plates (Sigma, St. Louis MO, USA). The products were then subjected to clean up by Sephadex gel column filtration using Sephadex® G-50 medium. Capillary electrophoresis was performed using ABI 3500® Genetic analyzer (Thermo Fisher Scientific Inc. USA). The electropherogram obtained from the ABI 3500 Genetic Analyzer is exported to the sequence analysis software, Codoncode® aligner program version 7.0. Comparison of sequences in the contig automatically identifies the variation in the sample sequence when aligned to a reference sequence, and located in the genome by Basic Local Alignment Search Tool (BLAST) [19] from National Center for Biotechnology Information (NCBI). Pathogenicity of variants was identified from different public databases such as dbSNP (NCBI, NIH USA), ClinVar (NCBI, NIH USA), and/or COSMIC database (Sanger Institute, UK). The pathogenicity of variants that were not reported in public databases or previous publications, were tested using computer-aided public accessible prediction tools such as MutationTaster [20], Polyphen [21] or Sorting Intolerant from Tolerant – SIFT- algorithm [22].

### Multiplex allele-specific real time PCR

Real Time PCR was performed using a CE/IVD approved commercial assay kit (TruPCR EGFR kit v2 from 3B BlackBio Biotech Ltd., Bhopal, India) for the detection of exon 19 deletion. In a 20 μl assay, 10 μl of 1X Multiplex Master mix and 5 μl of a probe mix was added along with 5 ng of template DNA (final concentration: 0.25 ng/μl). The probemix contains FAM labelled primer-probe specific for detection of exon 19 deletion and VIC labelled primer probe for exon 2 region of the *EGFR* gene, for detection of wildtype DNA as internal control. The assay conditions were as follows: Initial denaturation of 94 °C for 10 min, followed by 10 cycles of denaturation at 94 °C for 15 s and annealing at 68 °C for 30 s. This was followed by 40 cycles of denaturation at 94 °C for 15 s and annealing at 60 °C for 1 min. The assay was performed in LightCycler® 480 real-time PCR machine (Roche Molecular Diagnostics). The cycle threshold (C_t_) value for both FAM and VIC dyes were obtained for further analysis.

### HRM analysis

**Fig. 1:**
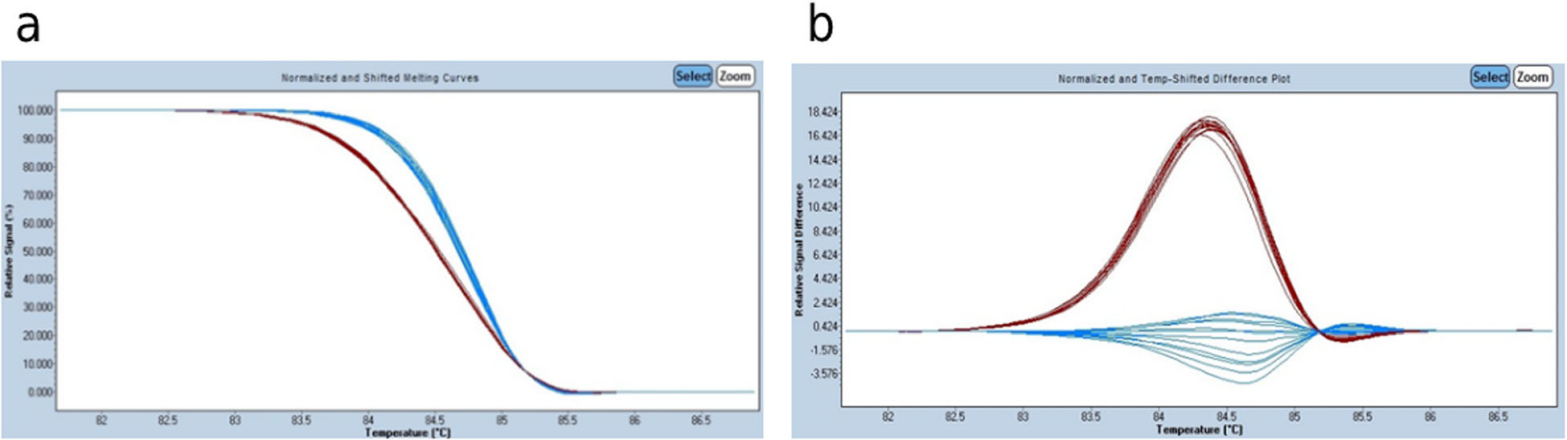
A typical HRM plot is depicted. The LightCycler® 480 Gene scanning software v1.5 automatically characterizes samples according to the HR melting pattern of the amplified DNA. **a** shows the normalized curve of fluorescence change in each small division of temperature change imparted onto the assay mix. Fluorescence of a double stranded DNA is taken as 100% and that of a fully denatured DNA is assigned as 0%. The temperature range at which the melting curve is analyzed is adjusted to align all the samples, according to the T_m_, to obtain uniform values of post- and pre-melt stages. Once the normalization is done, the software automatically differentiates samples that have a shifted melting curve from wild type. In the example above, the blue curves depict samples with wild-type DNA (wtDNA) while the red represents those with mutant DNA (mutDNA). The wtDNA has a slope (i.e. *d*F/*d*T) different from that of mutDNA. The second plot (**b**) is the “Difference plot”. In this graph, *d*F/*d*T (y-axis) is plotted against melting temperature range (x-axis), by subtracting the normalized shifted curve from a normalized base curve. The difference plot can visually differentiate even small changes in fluorescence intensity in unit temperature change. For this assay, 5 ng of total DNA was included to replicate previously established HRM assay condition

HRM was performed using the LightCycler® 480 real-time PCR and data was analyzed in LightCycler® 480 Gene scanning software 1.5, Windows version. In a typical HRM, during the melting phase, with increasing temperature, fluorescence decreases as the saturated dye detaches from the denatured amplicon DNA. The change in fluorescence per unit change in temperature (*d*F/*d*T) is plotted. Since the fluorescence intensity changes (HRM signal curve) for different samples have different end points, the data is normalized using the pre-melt and post-melt temperature ranges. Hence, each species of DNA is delineated according to the melting temperature. In the difference plot, the pre-assigned wildtype DNA is set as the baseline curve and the DNA from tumor tissues are plotted with different colors from that of the wildtype DNA. Significant deviation from the baseline curve is indicative of and assigned as mutant species by the software. The standard sensitivity is kept as 0.3 and as the sensitivity is increased, smaller deviations can be identified as mutant. The difference plot of heteroduplexes (where one strand is mutant and other strand is wildtype) shows maximum deviation from the wildtype species, while that of homoduplex mutant DNA shows intermediate deviation. Samples with an aberrant HRM curve are identified as mutant species. HRM results were compared with the results from SEQ for validating HRM analysis in the detection of *EGFR* mutation (Fig. 1).

### Statistical analysis

Statistical analysis was performed using IBM SPSS Windows version 20.0 software. Categorical variables are expressed using frequency and percentage. Diagnostic measures such as sensitivity, specificity and accuracy were calculated. McNemar’s test was used to test the statistical significance of the difference between HRM compared to standard test of SEQ.

## Results

### Mutation detection by SEQ

**Fig. 2:**
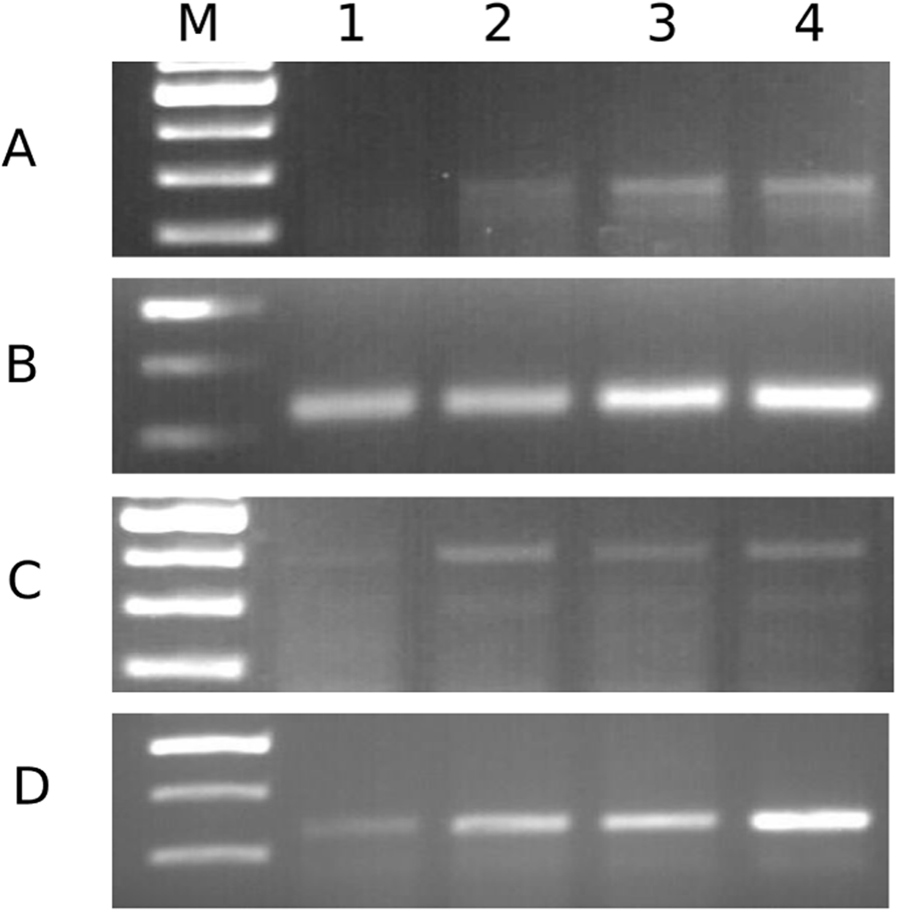
PCR products separated in a 2% agarose gel. Rows **a-d** depicts gradient PCR of exon 18, 19, 20 and 21 respectively. M denotes 100kbp ladder, lanes 1–4 represent the amplified products obtained at 55 °C, 58 °C, 60 °C and, 65 °C respectively. Although all exons were amplified at 65 °C, the maximum intensity for exon 20 was achieved at 58 °C (lane 2). In order to accommodate all the four exons in a single thermal cycler run, 58 °C was selected as the annealing temperature for the initial amplification for SEQ. Uncropped full images are given in Additional file 2: Figures S2-S4

Initial amplification PCR of exons 18–21 was standardized by performing a series of gradient PCRs to better accommodate assays for all four exons in a single thermal cycler setting. We found that at a lower annealing temperature than that described in previous studies, samples with low yield were better amplified, due to controlled reduction in specificity of the primers (see details in the discussion section). The standardization of the annealing temperature was performed using FFPE tissue-derived wildtype DNA (Fig. 2). At 58 °C, we observed satisfactory amplification for all four exons, following which all SEQ initial amplification PCR was conducted at this temperature.

With SEQ, 275 FFPE tumor samples received at MODL were screened for sequence variations in *EGFR* TKD (exons 18, 19, 20, and 21). About 27% of the samples (75/275) contained at least one pathogenic variant in any of the four exons. However, samples with well-differentiated adenocarcinoma of lung showed a 37% mutation occurrence rate. On the other hand, in poorly differentiated adenocarcinoma, mutation was detected in only 11% of the samples. Among the different exons of *EGFR* TKD which had mutation, exon 19 variations constituted about 69% (52/75) of the cases. Mutation in exon 21 was found in about 21% of the cases (16/75), and exons 18 and 20 variations were detected in one and eight samples, respectively.

### Limit of detection (LOD) of mutation fraction by SEQ

The DNA concentration for a typical SEQ was 2.5 ng/μl. In order to determine the lower limit of detection (LOD) of MAF, we used two commercially available standards of *EGFR* TKD mutants (mutDNA), 1) isolated DNA containing the exon 19 deletion, p.E746_A750del (Ex19_std) with 50% MAF (# HD251, Horizon Discovery Ltd., Ireland, UK), each vial contains 1 μg DNA in Tris-EDTA buffer (pH: 8.18, concentration: 50 ng/μl) with 50% mutant allele fraction of EGFR ΔE746-A750 (SNP ID: rs121913421) validated by digital droplet PCR, and 2) FFPE tissue containing the exon 21 mutation, L858R (Ex21_std) with 50% MAF (#HD130, Horizon Discovery Ltd., Ireland, UK) each vial contains one section FFPE cell pellet of human cell lines, 15-20 μm thick, with an approximate cell density of 3.5 × 10^5^ cells/section, containing roughly 400 ng total DNA with 50% mutant allele fraction of EGFR L858R (SNP ID: rs121434568) validated by digital droplet PCR.

**Fig. 3:**
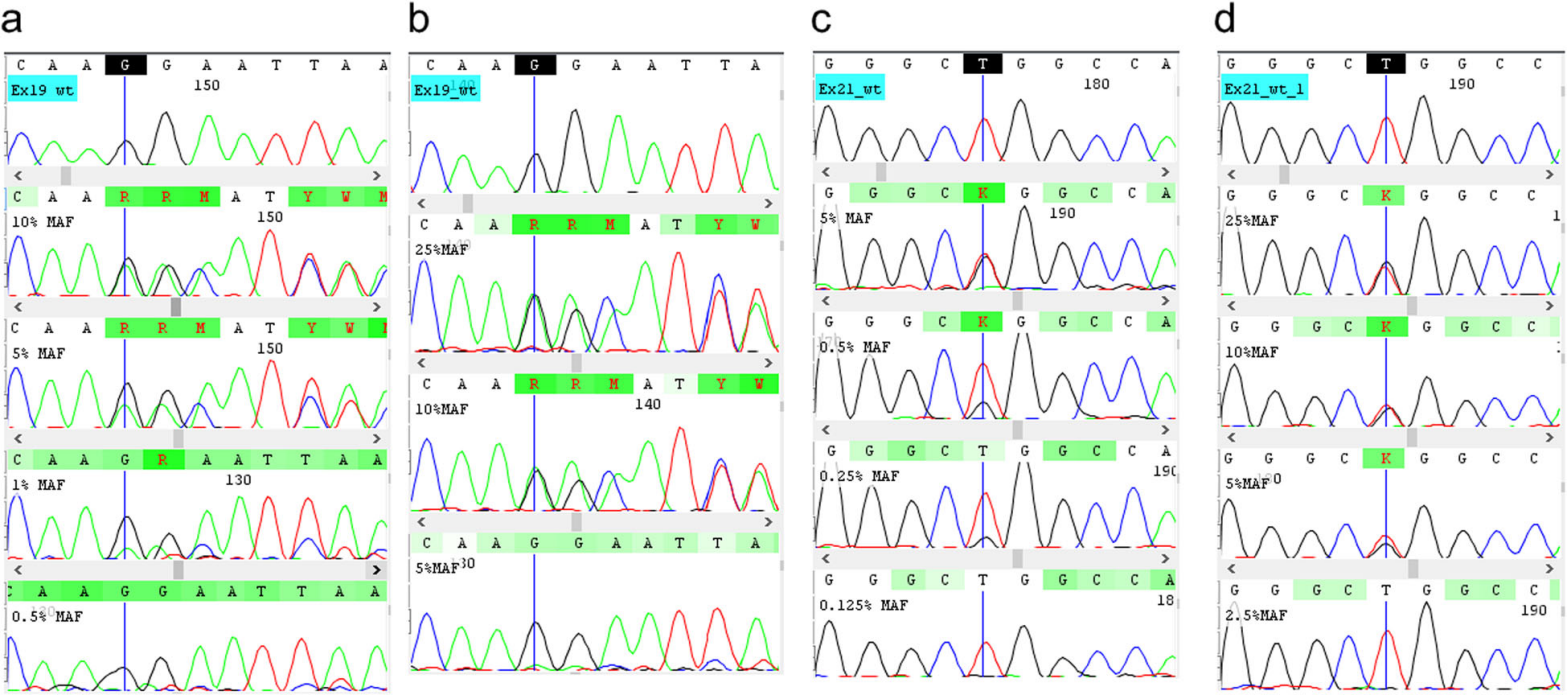
Chromatogram depicting LOD of MAF in Ex19_std and Ex21_std. **a:** Ex19_std with varying MAF 10%, 5%, 1% & 0.5% from top to bottom in a total DNA concentration of 2.5 ng/μl. **b:** Chromatogram of Ex19_std with varying MAF 25%, 10% & 5% from top to bottom in a total DNA concentration of 0.25 ng/μl. **c**: Chromatogram depicting Ex21_std with varying MAF 5%, 0.5%, 0.25%, 0.125% from top to bottom in a total DNA concentration of 2.5 ng/μl. **d:** Chromatogram of Ex21_std with varying MAF 25%, 10%, 5% & 2.5% from top to bottom for a total DNA concentration of 0.25 ng/μl. Note that the detection limits of Ex19_std & Ex21_std were 1% and 0.25% for 2.5 ng/μl assay and 10% and 5% for a 0.25 ng/μl assay

The LOD of MAF of Ex19_std and Ex21_std was separately determined by performing a series of assays with different total DNA concentrations ranging from 0.25 ng/μl (5 ng/assay of mutDNA + wtDNA) to 2.5 ng/μl (50 ng/assay of mutDNA + wtDNA). Figure 3a and b show the chromatograms of Ex19_std at two different total DNA concentrations (a: 2.5 ng/μl, b: 0.25 ng/μl), and Fig. 3c and d show the chromatograms of Ex21_std at two different total DNA concentrations (c: 2.5 ng/μl, d: 0.25 ng/μl). As seen from Fig. 3b and d, there was a sudden absence of the mutant peak at 5% MAF for Ex19_std and 2.5% MAF for Ex21_std at 0.25 ng/μl assay. However, in the 2.5 ng/μl, the mutant peak was not detected at 0.5% MAF for Ex19_std and 0.125% for Ex21_std. This shows that the LOD of MAF for a 0.25 ng/μl assay was 10% for Ex19_std, and 5% for Ex21_std for a 0.25 ng/μl assay. On the other hand, in a 2.5 ng/μl assay, lowest LOD for Ex19_std and Ex21_std were 1% and 0.25% respectively (Fig. 3a & c).

Since the origins of wtDNA and mutDNA were different, there can be a difference in amplification due to the quality of DNA. In order to address this, we performed SEQ with both wtDNA and mutDNA separately as well as in 1:1 mix. At 25% MAF, the wtDNA: mutDNA ratio was 1:1, for a total DNA concentration of 2.5 ng/μl. The peak heights of both wtDNA and mutDNA species were found to be equal, irrespective of cell-derived or tissue derived DNA (See Fig. 3b, d and Supplementary Fig. S1c). Moreover, the amplification peak heights of both tissue-derived wtDNA and cell-derived mutDNA, when sequenced separately were comparable as that of 1:1 mix (Fig. S1a and b). This shows that there was minimal difference between the quality of DNA obtained from both sources.

### Validation of SEQ LOD by real time PCR

**Fig. 4:**
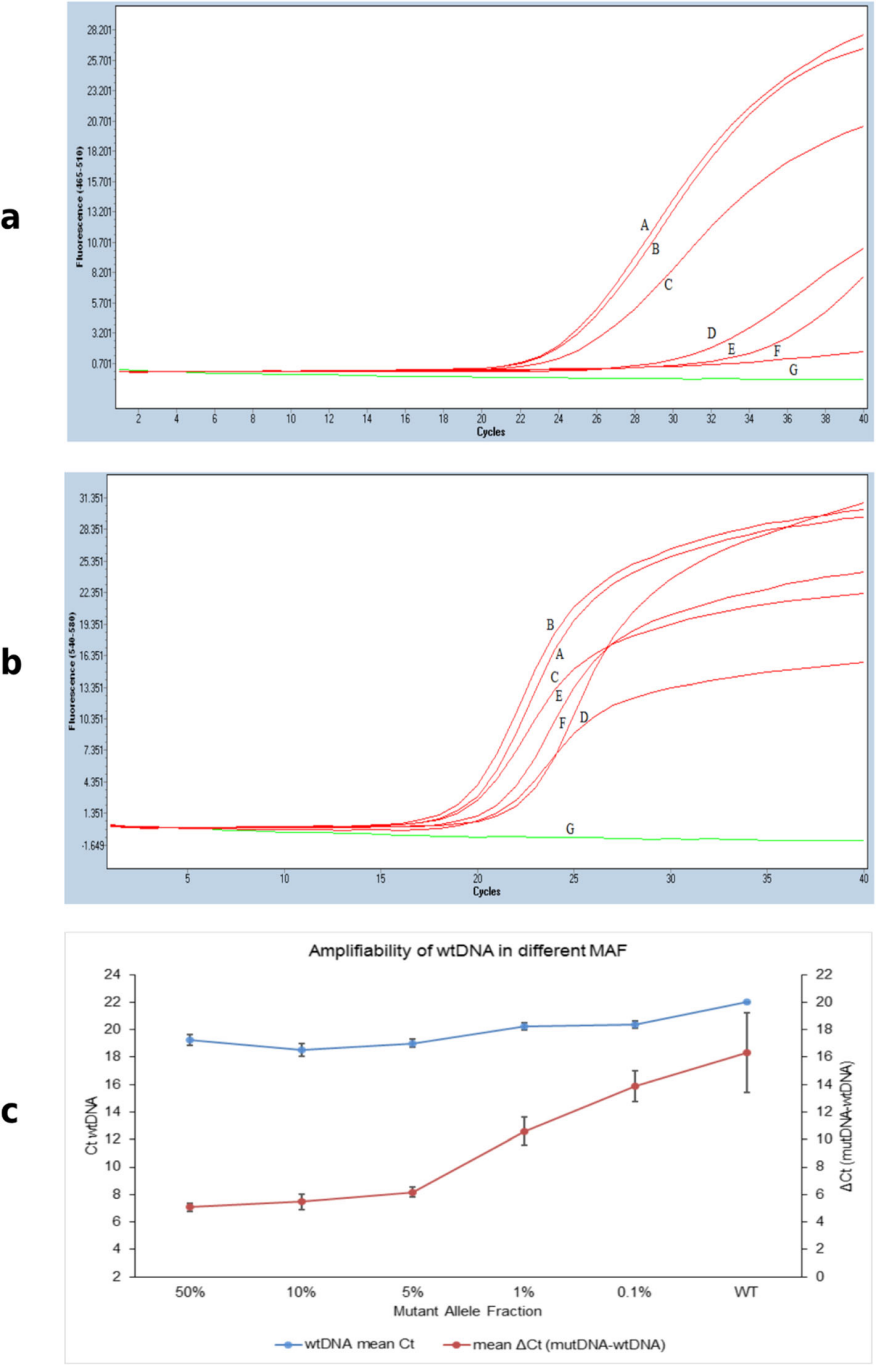
Allele-specific PCR with Ex19_std and wildtype DNA. Real time PCR with allele-specific amplification of Ex19_std and wildtype, in varying MAFs, in a total DNA concentration assay of 0.25 ng/μl. **a**: Ex19_std alone (50% MAF). **b**: 10% MAF of mutDNA in mixed DNA assay. Similarly, **c**: 5% MAF of mutDNA, **d**: 1% MAF, and **e**: 0.1% MAF in mixed DNA assays. **f**: wildtype DNA alone. **g**: non-template control. **a** and **b** are real-time PCR raw amplification profile of the mutant allele in the individual MAF assays depicted as fluorescent intensity (Y-axis) against cycle threshold (C_t_) values (X- axis). **a** is the profile of mutant DNA acquired in the FAM channel. **b** is the profile of wildtype DNA acquired in the VIC channel. **c:** Comparison of C_t_ values of wildtype DNA (left y-axis) in different MAF assays and the relationship between the C_t_ of wildtype and mutant alleles (right y-axis) obtained from the *Δ*C_t_ values for individual MAF assay (x- axis). Note that the *Δ*C_t_ values of upto 1% MAF were less than 12 and detected as mutant positive

With the intention of assessing both mutant and wildtype DNA amplification in mixed assays used in SEQ, multiplex real time PCR with allele-specific primer-probe for the detection of exon 19 deletion was performed. MAFs of 10%, 5%, 1% and 0.1% were assessed in triplicates in mixed DNA assays. Similarly, Ex19_std and wtDNA were also separately assessed. The multiplex allele-specific primer-probe is incorporated with two fluorescent dyes, FAM and VIC, detecting mutant and wildtype alleles, respectively. Accordingly, the amplifications of both the alleles were determined by the C_t_ values obtained for both dyes, respectively, in each assay. According to the manufacturer’s instruction, *Δ*C_t_ (The difference between C_t_ of mutant and C_t_ of wildtype) ≤ 12, indicates positive for the deletion of exon 19. The FAM C_t_ values showed wide variation across all MAFs, the lowest being detected in 50% MAF, while the highest in 0.1% MAF (Fig. 4 and Table 1). On the other hand, VIC C_t_ was comparable in all assays between all the MAFs (Fig. 4c). *Δ*C_t_ of all the MAFs, except 0.1% MAF, were detected as positive for exon 19 deletion (Table 1). Thus, the wildtype DNA as well as the mutant standard DNA were amplified in the mixed assays, thereby validating the lowest MAF detected in the identical assay using SEQ.

**Table 1:** Mean C_t_ obtained using allele-specific probe Real time PCR

MAF (%)	MutDNA (FAM) (mean ± sd)	wtDNA (VIC) (mean ± sd)	*Δ*C_t_ (mutDNA-wtDNA) (mean ± sd)
50	24.35 ± 0.32	19.27 ± 0.39	5.08 ± 0.29
10	24.03 ± 0.56	18.55 ± 0.47	5.47 ± 0.56
5	25.18 ± 0.27	19.01 ± 0.28	6.17 ± 0.36
1	30.83 ± 0.91	20.23 ± 0.28	10.6 ± 1.01
0.1	34.26 ± 0.89	20.37 ± 0.29	13.89 ± 1.15
WT	38.33 ± 2.89	22 ± 0.01	16.33 ± 2.89

### Standardization of HRM assay

**Fig. 5:**
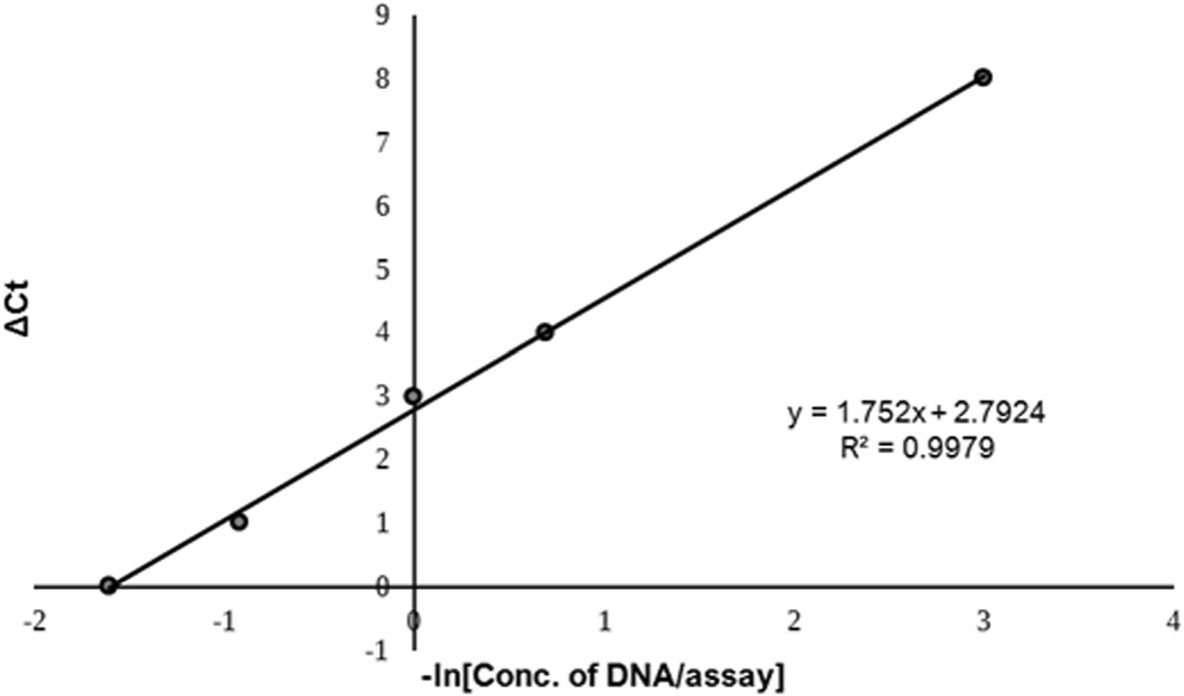
The relationship between the change in Ct value and concentration of DNA per assay. Log Dilution curve for 50% MAF sample showing C_t_ value difference (y-axis) to negative log of DNA concentration, −ln [DNA/assay], from 0.25 ng/μl to 0.5 pg/μl (x-axis). The trendline in the graph shows the correlation between -ln [DNA/assay] and *Δ*C_t_ value (*Δ*C_t_ value = C_t_ [at given DNA conc.] – C_t_ [maximum DNA conc.]). The inset of the graph shows the slope of the trendline and correlation (R^2^ value = 0.99). The relationship between *Δ*C_t_ value and concentration of DNA can be obtained from the slope of the graph

In order to assess the HRM detectability, a previous study by Do et al. included 5 ng of DNA per 20 μl assay (0.25 ng/μl final concentration) with 50% MAF [18]. We examined the LOD of initial DNA concentration by performing a dilution curve of mutDNA standard mixed with wtDNA in a real-time PCR assay using the same HRM conditions. Different mutDNA standard concentrations from 0.5 pg/μl to 0.25 ng/μl at 50% MAF were employed and assessed against C_t_ of real-time PCR assay. C_t_ value increases with decreasing concentration (Fig. 5). We found that the relative change in C_t_ values (*Δ*C_t_) was proportional to the natural logarithm of DNA concentration/assay as per the equation given below.

eq. 1 $$ \mathrm{Concentration}\kern0.5em \mathrm{of}\kern0.5em \mathrm{DNA}/\mathrm{assay}={e}^{-\left[\frac{\Delta \mathrm{Ct}-2.79}{1.752}\right]} $$

By applying the above equation, the saturation concentration of DNA for no change in *Δ*C_t_ value was ~ 0.249 ng/μl. Hence, the total DNA concentration/assay was fixed as 0.25 ng/μl for heteroduplex HRM analysis. This concentration is 10 times lower than the amount of DNA required per assay for SEQ.

### LOD of MAF by HRM

**Fig. 6:**
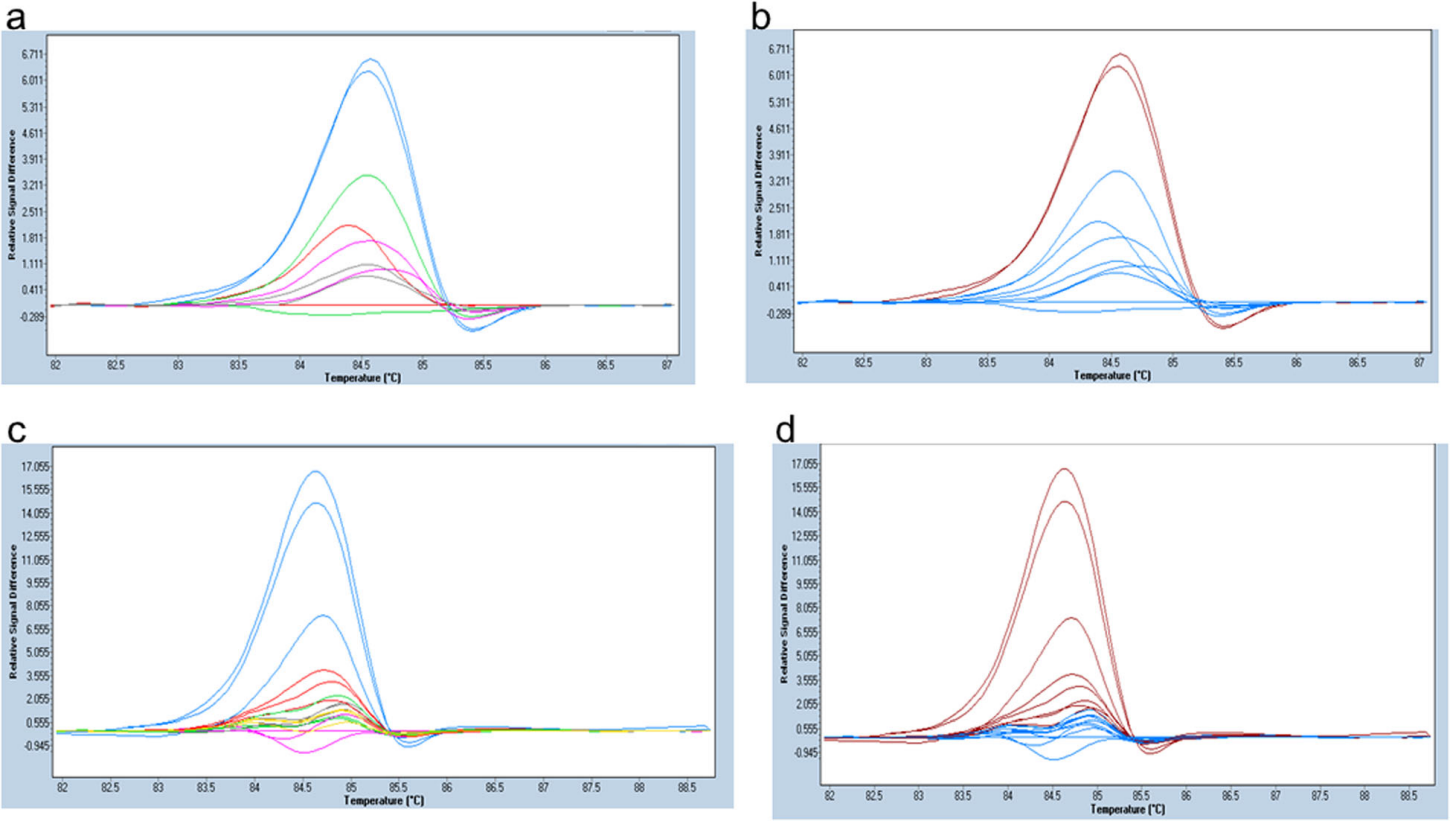
Difference plot depicting the different concentrations of Ex19_Std at 2.5 ng/μl & 0.25 ng/μl. **a** & **b** depicts HRM assays represented as difference plot, *d*F/*d*T (y-axis) is plotted against melting temperature range (x-axis). **a** depicts peaks with distinct colors annotated to different MAF, and **b** shows corresponding peaks detected as mutant or wildtype by HRM. Total DNA concentration of each peak was 2.5 ng/μl. MAF annotations in **a** are Blue (0.25%), Green (0.05%), Pink (0.025%), Grey (0.005%) and Red (wildtype). **b** shows the detection of mutant by HRM analysis in which Red is for Mutation positive and Blue for wildtype. Note that the detection by HRM as variant (shown as red peak in **b**) is only for the blue peak in **a**, which is 0.25%. **c** & **d** have a DNA concentration of 0.25 ng/μl. Samples with different MAF in **c** are annotated as Blue (50%), Red (5%), Grey (1%), Green (0.5%), Yellow (0.1%) and Pink wild-type. **d** shows the detection of variants by HRM. Red depicts Mutation positive and Blue depicts wildtype. Note that in **d**, HRM assay detected as mutant consistently in blue and red peaks in **c**. This means that HRM consistently detected variants up to 5% MAF

**Fig. 7:**
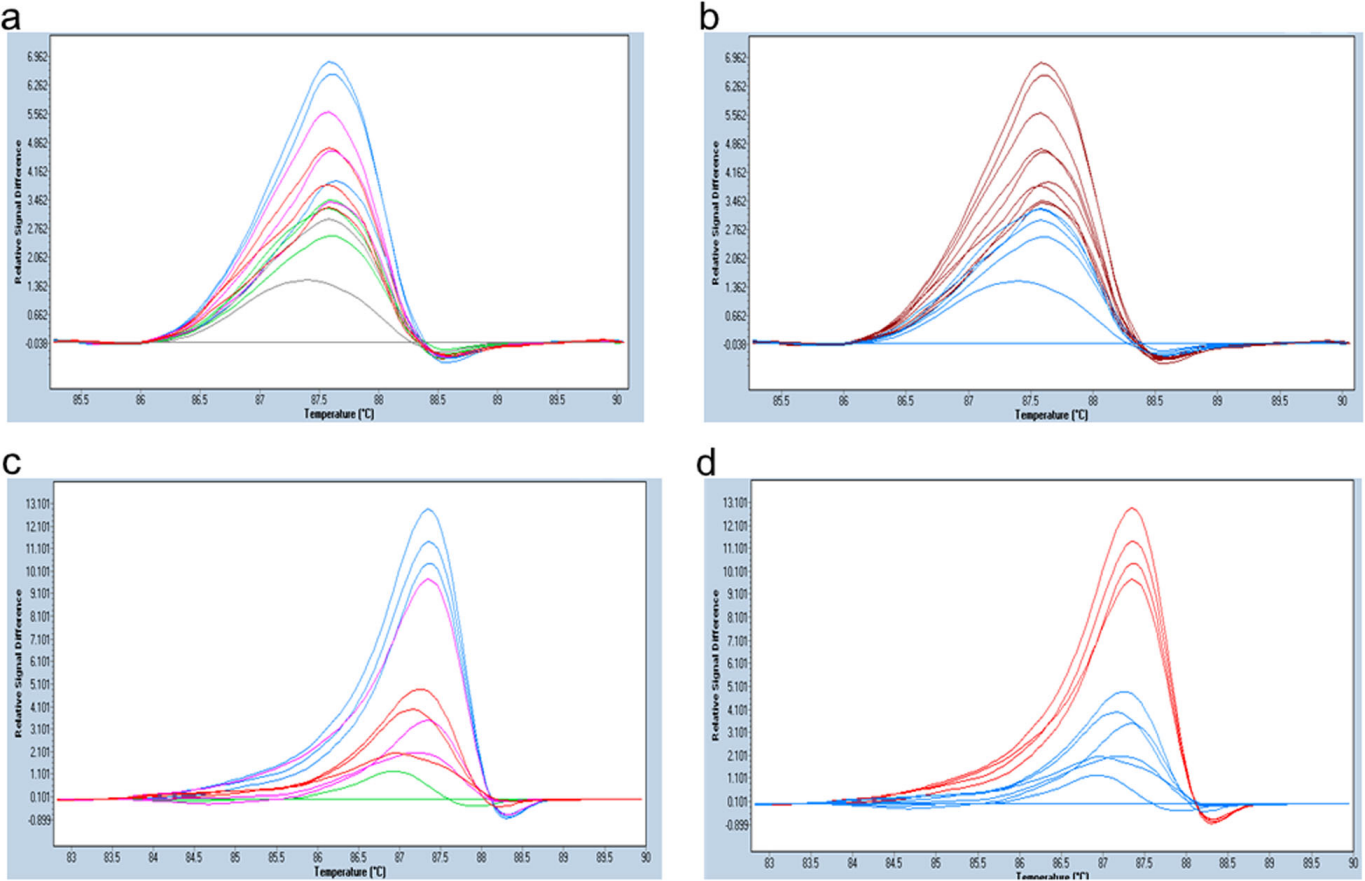
Difference plot depicting the different concentrations of Ex21_std at 2.5 ng/μl & 0.25 ng/μl. **a** and **b** depicts HRM assays represented as difference plot, *d*F/*d*T (y-axis) is plotted against melting temperature range (x-axis). **a** shows peaks with distinct colors annotated to different MAF, and **b** shows corresponding peaks detected as mutant or wildtype by HRM. Samples in both **a** & **b** have a total DNA concentration of 2.5 ng/μl. MAF annotations in **a** are Blue (1%), Pink (0.5%), Red (0.25%), Green (0.125%), and Grey (wildtype). **b** shows the detection of mutation (Red) or wildtype (Blue) by HRM. Note that the HRM detected mutants consistently (as red peak in **b**) in blue (1%), pink (0.5%) and red (0.25%) peaks in **a**. **c** & **d** have a DNA concentration of 0.25 ng/μl. **c** shows the difference plot depicting different MAF of Ex21_std as Blue (10%), Pink (5%), Red (2.5%) and Green (wild-type). **d** depicts the corresponding MAF as variant (Red) or wildtype (Blue). Note that the HRM consistently identified as variants (as red peak in **d**) in only blue peak (10% MAF)

In order to examine whether the LOD of MAF decreases with increasing concentration of total DNA (mutDNA+ wtDNA) per assay, we titrated mutDNA with wtDNA in a series of separate HRM assays containing varying total DNA concentrations. HRM MAF titration was conducted by incorporating decreasing MAF in a mixed DNA sample from 10% to 0.005% of both Ex19_std & Ex21_std, in an increasing total DNA concentration/assay from 0.25 ng/μl to 2.5 ng/μl (Figs. 6 and 7). For both the mutDNA standards, lowest LOD observed was 0.25% in a 2.5 ng/μl HRM assay, while in a 0.25 ng/μl assay, 5% MAF was the lowest LOD detected with the Ex19_std, and 10% with the Ex21_std.

### Comparative performance analysis

Of the 116 samples that were included in the study, 67 samples were mutation positive using the SEQ method (Table 2). Forty-seven of the 67 positive samples had deletion mutation in exon 19, and substitution mutations were detected in 15 and five samples in exon 21 (Table 3), and exon 20 respectively. None of the samples in this group were found to have a mutation in exon 18. Hence, the performance characteristics for exons 18 and 20 were not separately included in the comparative performance analysis. HRM positive samples were distributed as 72% positive and 28% negative in SEQ samples with the exon 19 mutation (Table 3). Furthermore, HRM positivity was distributed as 60% positive and 40% negative in SEQ samples with the exon 21 mutation (Table 3). The sensitivity of detection was 100% for both exon 19 and exon 21 mutations, while specificity was higher with the exon 21 variants compared to exon 19 variants (90% vs. 74%). McNemar’s comparison of these two methods was significant (*p* value < 0.01) for both the groups, and overall performance differences between these two methods. Overall specificity of HRM over SEQ was about 67% with 86% accuracy (Table 2). The positive predictive value (PPV) was 60% for exon 21 variants, about 72% for exon 19 variants, with an overall 80% PPV. However, the negative predictive value (NPV) was 100% in individual variant groups and overall values as well.

**Table 2:** Overall comparison of SEQ detected and HRM

HRM detected	SEQ Detected		***p*** value	Sensitivity	Specificity	PPV	NPV	Accuracy
	Positive (%)	Negative (%)						
Positive	67 (80.7)	16 (19.3)	< 0.001	100%	67.3%	80.7%	100%	86%
Negative	0 (0)	33 (100)

**Table 3:** Comparison of SEQ and HRM detected in Exon 19 and 21 mutation positive samples

HRM Detected	SEQ Detected		***p*** value	Sensitivity	Specificity	PPV	NPV	Accuracy
	Positive (%)	Negative (%)						
Positive (Exon 19)	47 (72.3)	18 (27.7)	< 0.001	100%	74%	72.3%	100%	84.4%
Negative (Exon 19)	0 (0)	51 (100)						
Positive (Exon 21)	15 (60)	10 (40)	0.002	100%	90.1%	60%	100%	91.3%
Negative (Exon 21)	0 (0)	91 (100)						

## Discussion

HRM is based on the principle of a small, yet definite, shift in denaturation temperature due to nucleotide base variation. The amplified DNA is subjected to stepwise heating to obtain controlled denaturation of the amplicons. HRM is highly specific to the species of DNA, with unique melting temperature, included in a heteroduplex assay. In this study, the somatic mutation detection efficiency of HRM was compared with SEQ. We have identified for the first time that the lowest MAF that can be detected in an HRM assay is 0.25%, irrespective of the type of somatic mutation in *EGFR* gene obtained from an FFPE tumor tissue. On the other hand, with similar amount of total DNA (50 ng), SEQ would require a minimum of 0.25% MAF for point mutation (p.L858R) and 1% MAF for exon 19 deletion for variant detection. We have also confirmed findings from earlier studies that, at 50% MAF, HRM can detect *EGFR* somatic variants with a total DNA concentration as low as 0.25 ng/μl. The LOD MAF of both the methods in this study is better than that of the previous two studies [23, 24]. The LOD MAF of both these methods for both types of mutation standards are comparable with an advantage for HRM with Ex19_std (SEQ∶ HRM = 1% ∶ 0.25%). This shows that HRM has higher sensitivity for the detection of exon 19 deletion mutations at a lower MAF, compared to SEQ. With multiplex real-time PCR, MAFs of upto 1% were detected in a 0.25 ng/μl mixed assay which is beyond the detection limit of SEQ and HRM, 10% and 5% respectively. Moreover, real-time PCR also validated the amplifiability of both mutant and wildtype DNA from two different sources used in the study.

A surprising observation in the current study is that the LOD of MAF by SEQ is 1% for exon 19 mutation and 0.25% for exon 21 mutation. This is the lowest detectable MAF by SEQ compared to previous studies and, on par with the detection limit of allele-specific real-time PCR [15, 25]. We have not adopted any selective enrichment methods such as amplification at optimized lower denaturation temperature or clamping methods. However, in order to achieve maximum possible, yet unbiased optimization, the conditions of initial PCR, specifically the denaturation and annealing temperatures, were standardized using gradient PCR (Fig. 2). Moreover, the optimization reactions were performed using wtDNA extracted from normal FFPE lung tissue. A method of PCR technology, where an optimized lower annealing temperature, when adopted could selectively induce an amplification bias and specifically inhibit amplification of major allele in a mixed DNA sample, was described in an earlier study using HRM [26], which was later patented [27]. The detection of lower MAF in a mixed (mutDNA + wtDNA) sample, in the current study, may be attributed to this modification in the annealing temperature. As a PCR enrichment method, Optimized Annealing Temperature PCR, now named as OAT-PCR, has not been widely used for somatic mutation analysis. We propose that this method can be utilized to selectively enrich and amplify minor mutant alleles from a mixed DNA sample, in an unbiased manner, irrespective of the sequence variation. This method may have a wider application in somatic mutation assessment via amplification-based sequencing methods like basic Sanger sequencing or advanced sequencing by synthesis methods. As this finding was beyond the scope of the current study, an extensive analysis is required to validate OAT-PCR for the detection of low MAF using SEQ.

In the series comparison study, as shown in Tables 2 and 3, HRM was able to detect all the positive samples that were detected by SEQ. However, there were several positive samples detected by HRM, especially the exon 19 variants, that were not detected by SEQ. Based on the results from the MAF detection limit of HRM, it can be concluded that the MAF of these samples may be below the detection range of SEQ, but within the limit of HRM, i.e. more than 0.25%, but lower than 10%. The sensitivity and NPV of HRM were found to be 100% compared to SEQ. On the other hand, the overall PPV was 67%. The “false-positive” samples of HRM were higher in the samples with potential exon 19 variants than exon 21 variants. In the comparative analysis of the patient samples, HRM specificity was lower in samples with exon 19 deletion than those with exon 21 mutation (74% vs. 90%). This is explained partially by the difference in LOD using these two methods for the Ex19_std. The possibility of false negativity could reduce with decreasing LOD, as in the case of HRM. Almost no difference in LOD of MAF was noted in exon 21 variants; hence, the chance of false negativity contributed by the difference in method may be very low. Therefore, higher specificity was observed with the exon 21 variants in comparative analysis.

## Conclusion

The performance comparison studies demonstrate that the NPV of HRM is very high due to high sensitivity as compared to SEQ. The NPV for both exon 19 and 21 is 100%, though PPV is about 70% for both exons. The overall accuracy is less than 90%, which suggests that HRM cannot be adopted as an independent diagnostic method for somatic mutation detection in FFPE samples. However, considering the widely varying MAF and DNA content in routine histopathological laboratory tumor tissues, HRM may be adopted as a good screening method for mutation detection, provided validation of HRM positive samples is conducted by methods such as SEQ or Next generation sequencing.

## Supplementary information

**Additional file 1: Figure S1.** SEQ Chromatogram of wtDNA and mutDNA. The panel of chromatogram of the comparative SEQ of two DNA species, FFPE lung tissue derived wild type DNA and cell derived mutant standard DNA (Exon 19 standard; ΔE746-A750) in a 2.5 ng/μl total DNA concentration assay. The first panel (S1a) is the SEQ data of wtDNA obtained from FFPE lung tissue. The second panel (S1b) is the SEQ data of standard cell-derived mutDNA obtained from Horizon, and the third panel marked “25% MAF” is the SEQ data of mixed DNA from both FFPE wtDNA and cell-derived standard mutDNA (1:1).

**Additional file 2: Figures S2-S4.** Uncropped raw gel images of Fig. 2. Uncropped raw gel images of Fig. 2 from three different sets of gradient PCR. **Figure S2** is the uncropped full image of Fig. 2a and c. **Figure S3** is the uncropped image of Fig. 2b and **Figure S4** is the uncropped image of Fig. 2d. Details are given in the legends.

## Data Availability

All data generated or analyzed during this study are included in this published article. The raw data from this study are available at the Open Science Forum Repository. Data can be accessed openly from the link: http://bit.ly/HRMtoSanger.

## Abbreviations

EGFR: Epidermal Growth Factor Receptor
HRM: High Resolution Melting Analysis
LOD: Limit of Detection
MAF: Mutant Allele Fraction
MODL: Molecular Oncology Diagnostic Laboratory
NSCLC: Non-small cell lung cancer
AIMS: Amrita Institute of Medical Sciences
PCR: Polymerase Chain Reaction
wtDNA: Wildtype DNA
mutDNA: Mutant DNA
SEQ: Sanger sequencing
TKD: Tyrosine Kinase Domain

## References

[1] Vogelstein B, Papadopoulos N, Velculescu VE, Zhou S, Diaz LA, Kinzler KW (2013). Cancer genome landscapes. Science.

[2] Nicholson RI, Gee JM, Harper ME (2001). EGFR and cancer prognosis. Eur J Cancer.

[3] Paez JG, Janne PA, Lee JC, Tracy S, Greulich H, Gabriel S, Herman P, Kaye FJ, Lindeman N, Boggon TJ (2004). EGFR mutations in lung cancer: correlation with clinical response to gefitinib therapy. Science.

[4] Pao W, Miller V, Zakowski M, Doherty J, Politi K, Sarkaria I, Singh B, Heelan R, Rusch V, Fulton L (2004). EGF receptor gene mutations are common in lung cancers from "never smokers" and are associated with sensitivity of tumors to gefitinib and erlotinib. Proc Natl Acad Sci U S A.

[5] Lynch TJ, Bell DW, Sordella R, Gurubhagavatula S, Okimoto RA, Brannigan BW, Harris PL, Haserlat SM, Supko JG, Haluska FG (2004). Activating mutations in the epidermal growth factor receptor underlying responsiveness of non-small-cell lung cancer to gefitinib. N Engl J Med.

[6] Lindeman NI, Cagle PT, Aisner DL, Arcila ME, Beasley MB, Bernicker EH, Colasacco C, Dacic S, Hirsch FR, Kerr K (2018). Updated molecular testing guideline for the selection of lung Cancer patients for treatment with targeted tyrosine kinase inhibitors: guideline from the College of American Pathologists, the International Association for the Study of Lung Cancer, and the Association for Molecular Pathology. J Thorac Oncol.

[7] Lindeman NI, Cagle PT, Aisner DL, Arcila ME, Beasley MB, Bernicker EH, Colasacco C, Dacic S, Hirsch FR, Kerr K (2018). Updated molecular testing guideline for the selection of lung Cancer patients for treatment with targeted tyrosine kinase inhibitors: guideline from the College of American Pathologists, the International Association for the Study of Lung Cancer, and the Association for Molecular Pathology. Arch Pathol Lab Med.

[8] Cohen V, Agulnik JS, Jarry J, Batist G, Small D, Kreisman H, Tejada NA, Miller WH, Chong G (2006). Evaluation of denaturing high-performance liquid chromatography as a rapid detection method for identification of epidermal growth factor receptor mutations in nonsmall-cell lung cancer. Cancer.

[9] Janne PA, Borras AM, Kuang Y, Rogers AM, Joshi VA, Liyanage H, Lindeman N, Lee JC, Halmos B, Maher EA (2006). A rapid and sensitive enzymatic method for epidermal growth factor receptor mutation screening. Clin Cancer Res.

[10] Lipsky RH, Mazzanti CM, Rudolph JG, Xu K, Vyas G, Bozak D, Radel MQ, Goldman D (2001). DNA melting analysis for detection of single nucleotide polymorphisms. Clin Chem.

[11] Li J, Wang L, Mamon H, Kulke MH, Berbeco R, Makrigiorgos GM (2008). Replacing PCR with COLD-PCR enriches variant DNA sequences and redefines the sensitivity of genetic testing. Nat Med.

[12] Helmy M, Awad M, Mosa KA (2016). Limited resources of genome sequencing in developing countries: challenges and solutions. Appl Transl Genom.

[13] Borras E, Jurado I, Hernan I, Gamundi MJ, Dias M, Marti I, Mane B, Arcusa A, Agundez JA, Blanca M (2011). Clinical pharmacogenomic testing of KRAS, BRAF and EGFR mutations by high resolution melting analysis and ultra-deep pyrosequencing. BMC Cancer.

[14] Fukui T, Ohe Y, Tsuta K, Furuta K, Sakamoto H, Takano T, Nokihara H, Yamamoto N, Sekine I, Kunitoh H (2008). Prospective study of the accuracy of EGFR mutational analysis by high-resolution melting analysis in small samples obtained from patients with non-small cell lung cancer. Clin Cancer Res.

[15] Pichler M, Balic M, Stadelmeyer E, Ausch C, Wild M, Guelly C, Bauernhofer T, Samonigg H, Hoefler G, Dandachi N (2009). Evaluation of high-resolution melting analysis as a diagnostic tool to detect the BRAF V600E mutation in colorectal tumors. J Mol Diagn.

[16] Takano T, Ohe Y, Tsuta K, Fukui T, Sakamoto H, Yoshida T, Tateishi U, Nokihara H, Yamamoto N, Sekine I, et al. Epidermal growth factor receptor mutation detection using high-resolution melting analysis predicts outcomes in patients with advanced non small cell lung cancer treated with gefitinib. Clin Cancer Res. 2007;13. 10.1158/1078-0432.CCR-07-0627 17875767

[17] Nomoto K, Tsuta K, Takano T, Fukui T, Fukui T, Yokozawa K, Sakamoto H, Yoshida T, Maeshima AM, Shibata T (2006). Detection of EGFR mutations in archived cytologic specimens of non-small cell lung cancer using high-resolution melting analysis. Am J Clin Pathol.

[18] Do H, Krypuy M, Mitchell PL, Fox SB, Dobrovic A (2008). High resolution melting analysis for rapid and sensitive EGFR and KRAS mutation detection in formalin fixed paraffin embedded biopsies. BMC Cancer.

[19] Altschul SF, Gish W, Miller W, Myers EW, Lipman DJ (1990). Basic local alignment search tool. J Mol Biol.

[20] Schwarz JM, Cooper DN, Schuelke M, Seelow D (2014). MutationTaster2: mutation prediction for the deep-sequencing age. Nat Methods.

[21] Adzhubei IA, Schmidt S, Peshkin L, Ramensky VE, Gerasimova A, Bork P, Kondrashov AS, Sunyaev SR (2010). A method and server for predicting damaging missense mutations. Nat Methods.

[22] Sim NL, Kumar P, Hu J, Henikoff S, Schneider G, Ng PC (2012). SIFT web server: predicting effects of amino acid substitutions on proteins. Nucleic Aacids Res.

[23] Tsiatis AC, Norris-Kirby A, Rich RG, Hafez MJ, Gocke CD, Eshleman JR, Murphy KM (2010). Comparison of sanger sequencing, pyrosequencing, and melting curve analysis for the detection of KRAS mutations: diagnostic and clinical implications. J Mol Diagn.

[24] Lin MT, Mosier SL, Thiess M, Beierl KF, Debeljak M, Tseng LH, Chen G, Yegnasubramanian S, Ho H, Cope L (2014). Clinical validation of KRAS, BRAF, and EGFR mutation detection using next-generation sequencing. Am J Clin Pathol.

[25] Angulo B, Garcia-Garcia E, Martinez R, Suarez-Gauthier A, Conde E, Hidalgo M, Lopez-Rios F (2010). A commercial real-time PCR kit provides greater sensitivity than direct sequencing to detect KRAS mutations: a morphology-based approach in colorectal carcinoma. J Mol Diagn.

[26] Idaho Technology I (2008). Mutant allele amplification Bias using rapid cycle-real time PCR and hi-res melting® with LunaProbes™ on the LightScanner® 32. BioTechniques.

[27] Mckinney JT, Zhou L, Palais RA (2016). Allele Amplification Bias. Edited by Office USPaT. United States of America: Biofire Defense, LLC (Salt Lake City, UT, US), University of Utah Research Foundation (Salt Lake City, UT, US).

