# Supplementary figures and images for: Somatic mutation detection efficiency in EGFR: a comparison between high resolution melting analysis and Sanger sequencing
Source: BMC Cancer. 2020 Sep 22;20:902. doi: 10.1186/s12885-020-07411-1 (PMC7510081; doi:10.1186/s12885-020-07411-1)

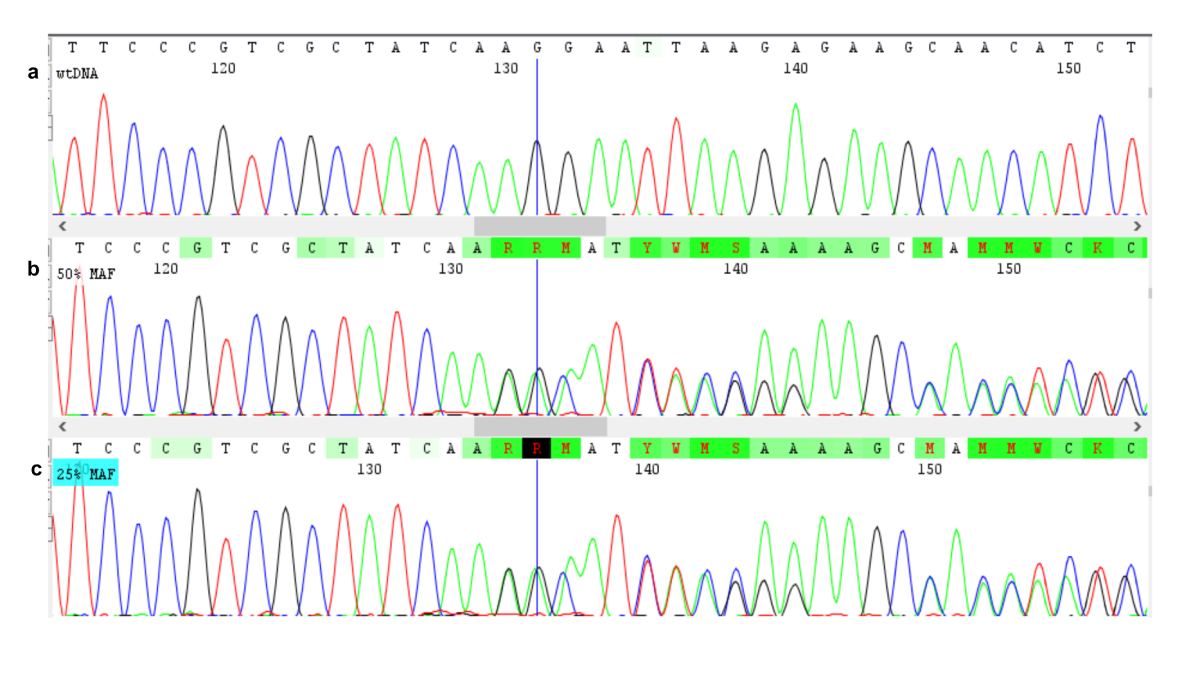

Supplement: Supplementary file 1 — Additional file 1: Figure S1. SEQ Chromatogram of wtDNA and mutDNA. The panel of chromatogram of the comparative SEQ of two DNA species, FFPE lung tissue derived wild type DNA and cell derived mutant standard DNA (Exon 19 standard; ΔE746-A750) in a 2.5 ng/μl total DNA concentration assay. The first panel (S1a) is the SEQ data of wtDNA obtained from FFPE lung tissue. The second panel (S1b) is the SEQ data of standard cell-derived mutDNA obtained from Horizon, and the third panel marked “25% MAF” is the SEQ data of mixed DNA from both FFPE wtDNA and cell-derived standard mutDNA (1:1). [file 12885_2020_7411_MOESM1_ESM.tiff]
