# Supplementary material for: Somatic mutation detection efficiency in EGFR: a comparison between high resolution melting analysis and Sanger sequencing
Source: BMC Cancer. 2020 Sep 22;20:902. doi: 10.1186/s12885-020-07411-1 (PMC7510081; doi:10.1186/s12885-020-07411-1)
Supplement: Supplementary file 2 — Additional file 2: Figures S2-S4. Uncropped raw gel images of Fig. 2. Uncropped raw gel images of Fig. 2 from three different sets of gradient PCR. Figure S2 is the uncropped full image of Fig. 2a and c. Figure S3 is the uncropped image of Fig. 2b and Figure S4 is the uncropped image of Fig. 2d. Details are given in the legends. [file 12885_2020_7411_MOESM2_ESM.docx]

**Additional File 2: Uncropped gel images.**

**Figure S2: Raw gel image of Figure 2 (Row A & C)**


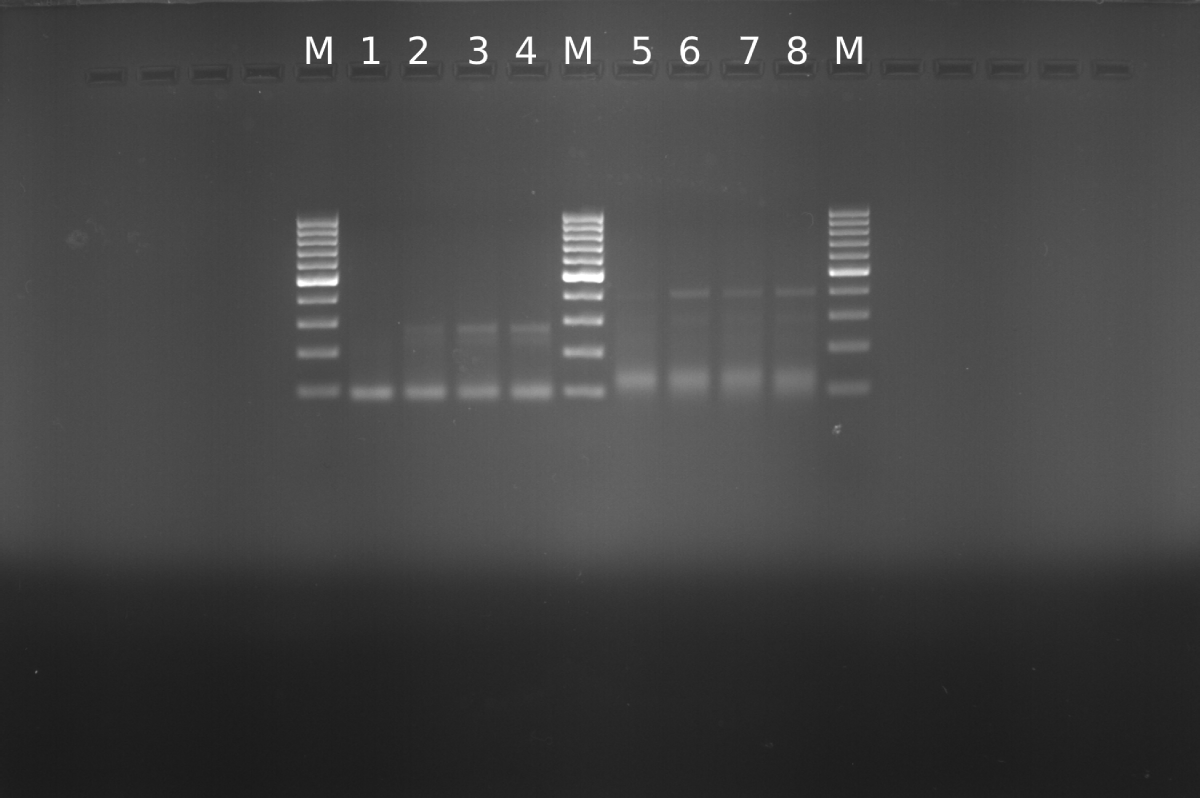


**Figure S2: M** denotes 100kbp ladder, lanes **1-4** represent the amplified products obtained at 55℃, 58℃, 60℃ and, 65℃ respectively for Exon 18. Lanes **5-8** represent the amplified products obtained at 55℃, 58℃, 60℃ and, 65℃ respectively for Exon 20.

**Figure S3: Raw gel image of Figure 2 (Row B)**


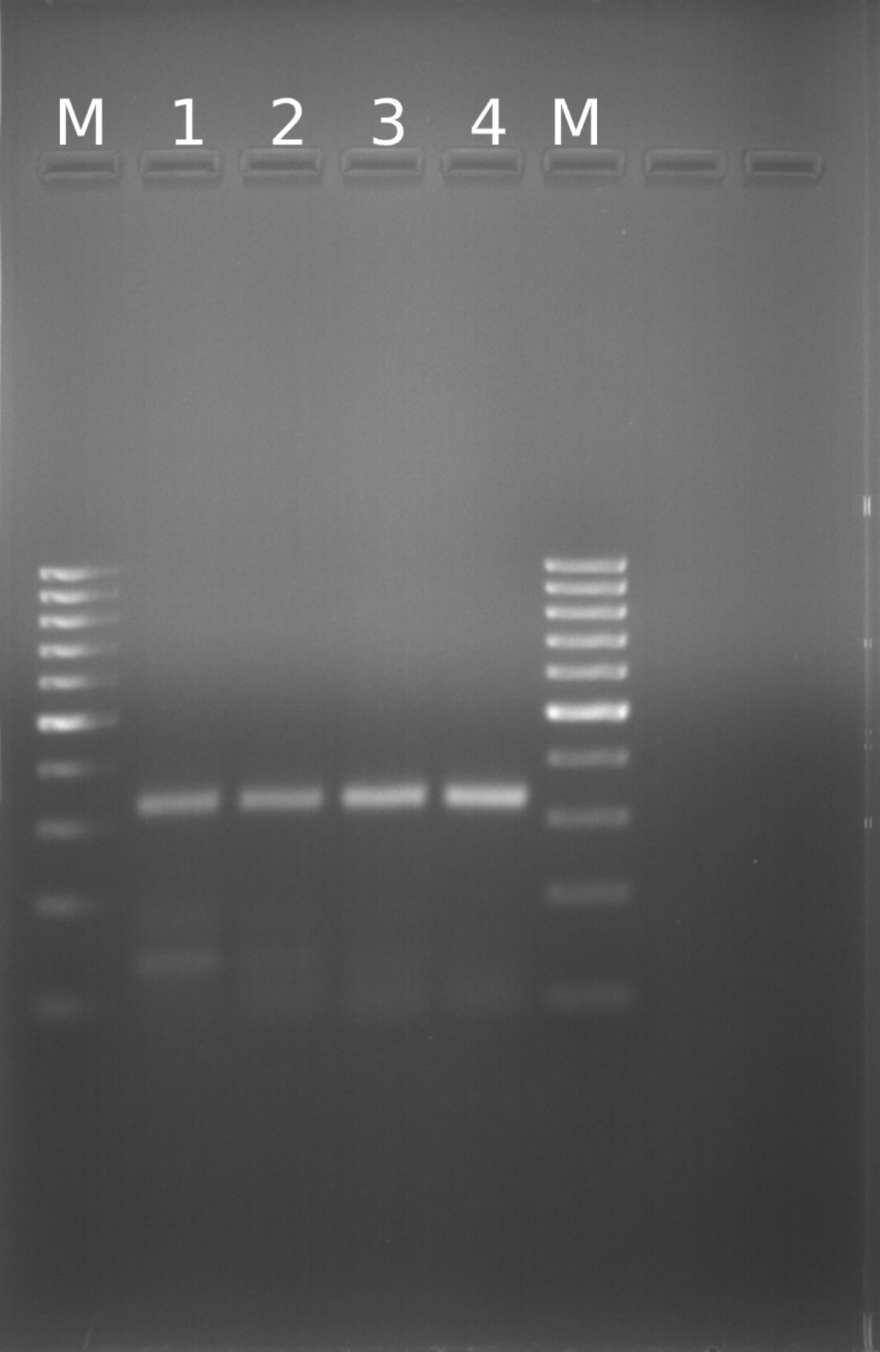


**Figure S3: M** denotes 100kbp ladder, lanes **1-4** represent the amplified products obtained at 55℃, 58℃, 60℃ and, 65℃ respectively for Exon 19.

**Figure S4: Raw gel image of Figure 2 (Row D)**


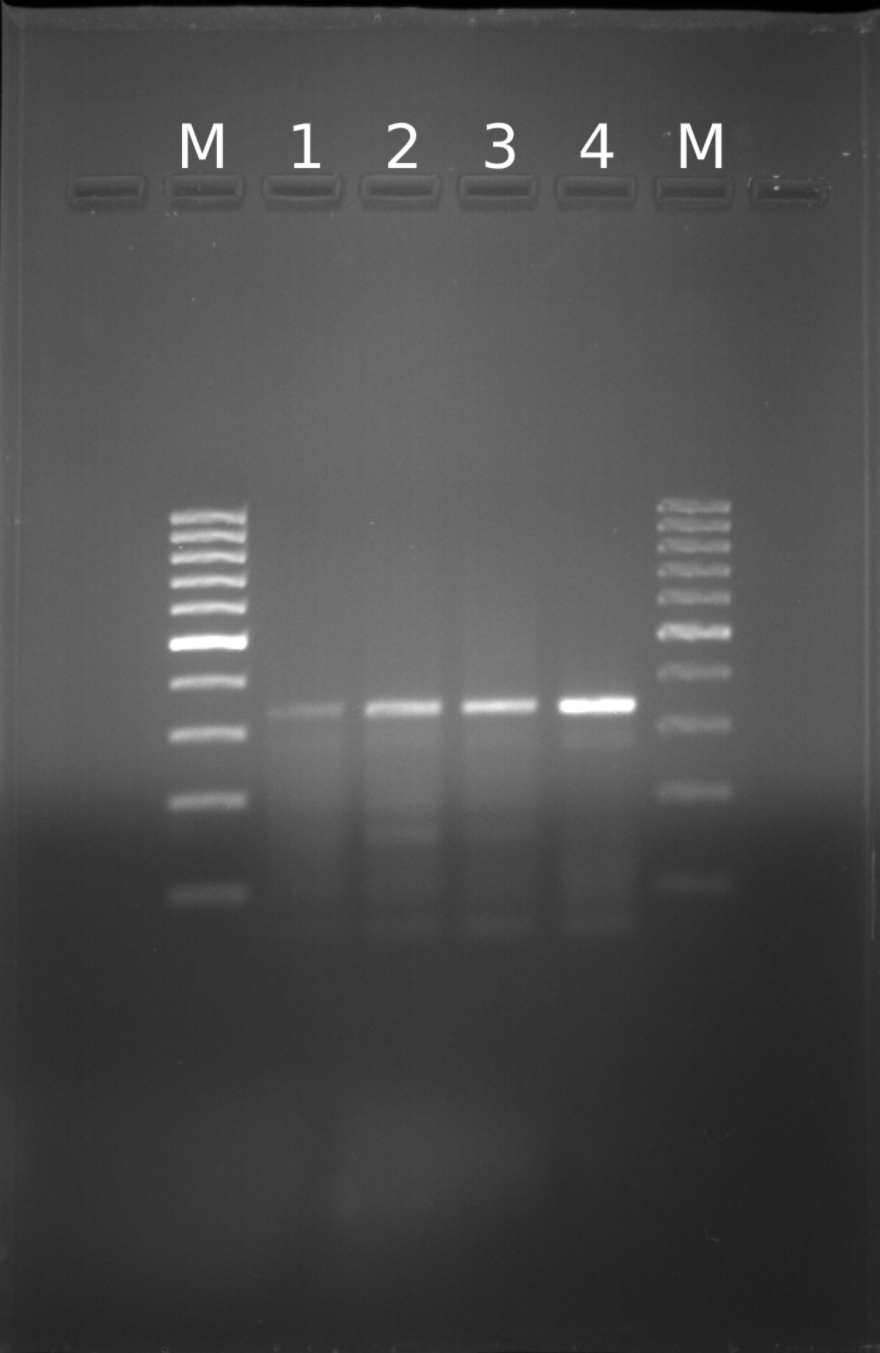


**Figure S4: M** denotes 100kbp ladder, lanes **1-4** represent the amplified products obtained at 55℃, 58℃, 60℃ and, 65℃ respectively for Exon 21. See figure 6 and legend for detailed description of the image.
